# Imaging and Therapy of Tumors Based on Neutrophil Extracellular Traps

**DOI:** 10.1002/smsc.202400212

**Published:** 2024-06-21

**Authors:** Yongwei Hao, Dalin Liu, Kaiyuan Wang, Qian Liu, Hongli Chen, Shenglu Ji, Dan Ding

**Affiliations:** ^1^ School of Life Sciences and Technology Xinxiang Medical University 601 Jinsui Avenue Xinxiang 453003 China; ^2^ Department of Urology Tianjin First Central Hospital Tianjin 300192 China; ^3^ Frontiers Science Center for Cell Responses State Key Laboratory of Medicinal Chemical Biology Key Laboratory of Bioactive Materials Ministry of Education College of Life Sciences Nankai University Tianjin 300071 China

**Keywords:** cancer therapies, DNase‐1, fluorescent probes, nanomaterials, neutrophil extracellular traps

## Abstract

Neutrophil extracellular traps (NETs) formed by neutrophils are netlike scaffolds that mainly contain DNA and a variety of granule proteins. Many stimuli can lead to the NET formation through independent molecular pathways. Clinically, the abundance of NETs is correlated with poor tumor prognosis. The biological actions of NETs are complex and diverse, including promoting tumor progression, awakening the dormant cancer cells, and resulting in immunosuppression in support of tumor growth and metastasis. Therefore, NET‐associated pathological processes provide an important clue for both diagnostic imaging and alternative therapies for many kinds of cancers. In recent years, scientists’ efforts have focused on developing novel imaging probes to visualize NETs and therapeutic strategies by degrading NETs or inhibiting its formation to block their pro‐tumoral functions. In this review, the development and evaluation of NETs‐targeted imaging and intervention progress for tumor therapy are focused on.

## Graphical abstract

In this review, an overview of emerging technologies in the field of imaging and therapy of tumors based on neutrophil extracellular traps (NETs), including imaging of NETs and pharmacological modulation of NETs for tumor therapy, is provided. The prospects and challenges of NETs in emerging fields are also discussed.

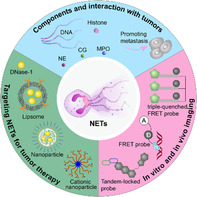

## 1 Introduction

During the past years, many approaches (e.g., chemotherapy, immunotherapy, and physical therapy) were developed for cancer therapy.^[^ ^1^ ^]^ However, the underlying mechanisms of tumor development, therapy resistance, and metastasis are not so far clear, which can reduce the effectiveness of treatment.^[^ ^2^ ^]^ To date, much attention has been paid to the tumor microenvironment (TME) to develop new strategies to remove primary and secondary tumors, and to inhibit further metastasis events.

**Figure 1:**
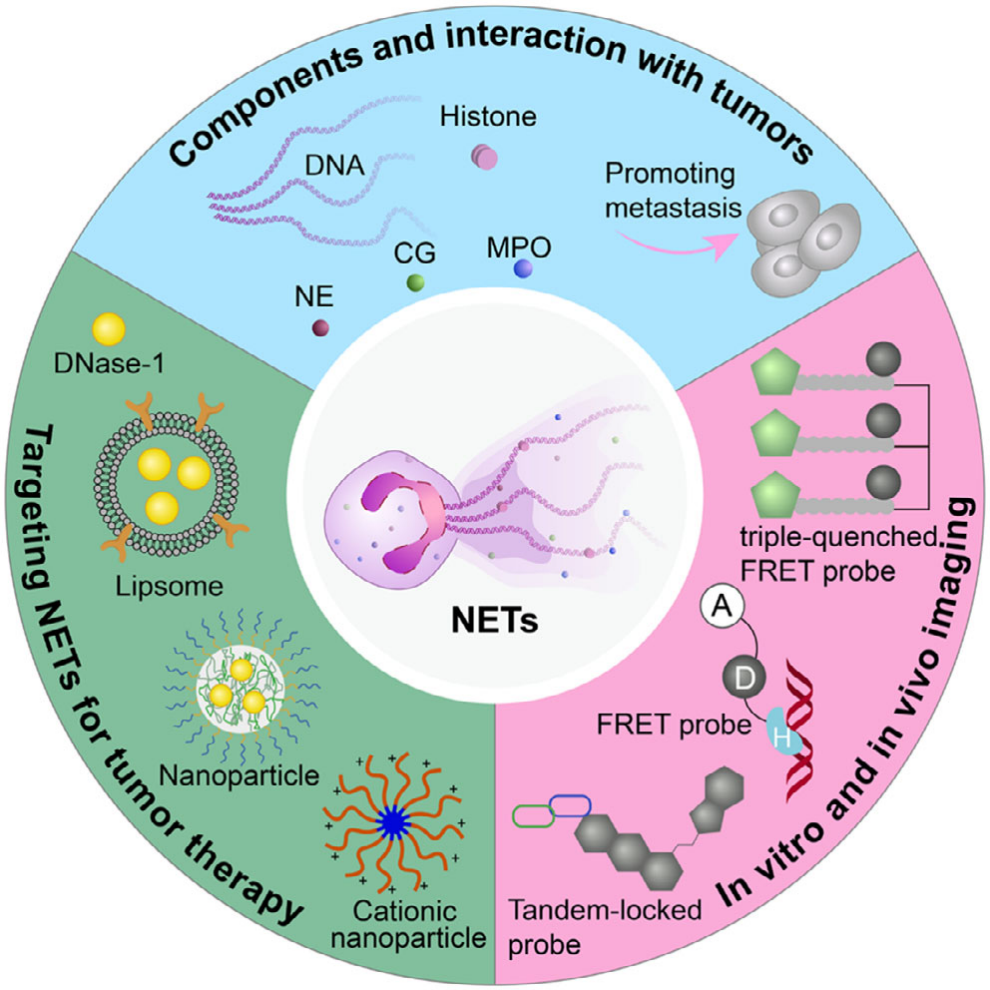
Schematic illustration of neutrophil extracellular traps (NETs) in tumors as well as the strategies of visualization and targeting NETs for cancer therapy.

Neutrophils are the main component of the innate immune system.^[^ ^3^ ^]^ They are also the primary responders during acute inflammation. Compared to the percentage of neutrophils in circulating leukocytes (around 10%–25%) in mice, human neutrophils occupy more than half the number of circulating leukocytes (around 50%–70%).^[^ ^4^ ^]^ In general, mature neutrophils in circulation have a segmented nucleus with a diameter of 7–10 μm.^[^ ^5^ ^]^ Traditionally, they are considered to be short‐lived immune cells, and their circulating half‐life is only 6–8 h in humans and mice.^[^ ^6^ ^]^ Interestingly, neutrophil extracellular traps (NETs) have been found as a special fate of neutrophils, which create a reticulated DNA fibers structure decorated with histones and granulins proteins, such as myeloperoxidase, neutrophil elastase (NE), matrix metalloproteinase 9 (MMP‐9), and others (**Figure** **1**).^[^ ^7^ ^]^

According to mounting data in clinic, NETs play an important role in infection,^[^ ^8^ ^]^ autoimmunity,^[^ ^9^ ^]^ and cancer.^[^ ^10^ ^]^ Of note, NETs were demonstrated to be associated with tumor progression, metastasis, and therapy resistance. The presence of NETs associated with tumors was first revealed in Ewing sarcoma (EWS) patients, and tumor‐associated NETs could lead to dismal prognoses.^[^ ^11^ ^]^ So far, many researches have revealed that NETs have pro‐tumorigenic properties in many kinds of tumors, including breast cancer,^[^ ^12^ ^]^ lung cancer,^[^ ^13^ ^]^ gastric cancer,^[^ ^14^ ^]^ colorectal cancer (CRC),^[^ ^15^ ^]^ head and neck squamous cell carcinoma,^[^ ^16^ ^]^ pancreatic cancer,^[^ ^17^ ^]^ bladder cancer,^[^ ^18^ ^]^ ovarian cancer,^[^ ^19^ ^]^ and EWS.^[^ ^20^ ^]^ To date, inflammation in infection, neutrophils infiltration, hypoxic microenvironment formation, immune microenvironment change, and impaired NETs degradation are reported to be involved in the NETs formation in tumors.^[^ ^21^ ^]^ Specially, the NETs formation is also correlated to tumors behavior. For example, many inflammatory molecules released by tumor cells such as CXCL1, CXCL2, CXCL8, or mesothelin secretion have the ability to induce intratumoral NETs.^[^ ^22^ ^]^ Moreover, another research have demonstrated that tumor extracellular vesicles could induce NETs to promote lymph node metastasis.^[^ ^23^ ^]^ However, the NETs production in tumor development is complex and some mechanisms are still unclear. Therefore, unraveling the interplay between NETs formation and tumor development will help better understand the pathology and treatment of many tumors.

Given the role of NETs in tumor progression, metastasis, and therapy resistance, developing methods for imaging and intervention of NETs is important in clinical and research applications. This article first summarized the historical timeline of advances in the bidirectional interplay between cancer and NETs, and then reviewed the methods for imaging and therapy of NETs for inhibiting tumor growth and tumor metastasis (Figure 1).

## 2 The NETs at a Glance

**Figure 2:**
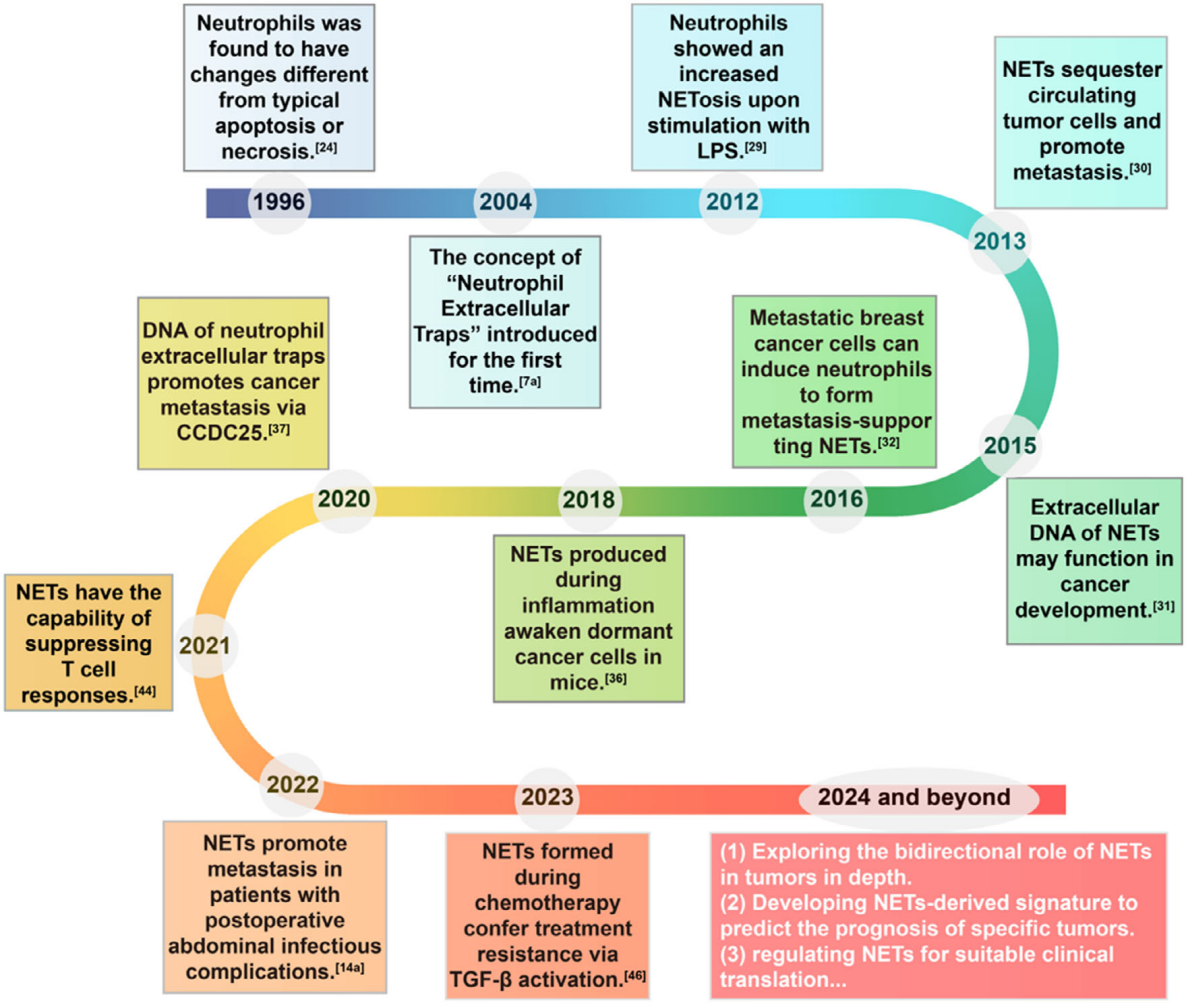
Historical timeline of significant advances in the bidirectional interplay between cancers and NETs.

The earliest study of NETs can be traced back to 1996 (**Figure** **2**). At that time, it was discovered that human neutrophils died quickly when they were exposed to phorbol 12‐myristate 13‐acetate (PMA) while this was distinct from typical apoptosis or necrosis because of the beginning morphological changes in the nucleus.^[^ ^24^ ^]^ In 2004, Volker Brinkmann et al. formally proposed “neutrophil extracellular traps” to describe this cell pathway based on the morphological changes.^[7a]^ In the beginning, the release of NETs was only identified as a mechanism of bacterial killing. Subsequently, the classification of NETs formation was gradually identified. Nicotinamide adenine dinucleotide phosphate (NADPH)‐oxidase (Nox)‐dependent NETs formation was initially believed to be a suicidal form.^[^ ^25^ ^]^ However, other researchers have demonstrated that Nox‐independent NETs also occurred through calcium (Ca^2+^) influx and mitochondrial reactive oxygen species (ROS) generation.^[^ ^26^ ^]^ Subsequently, another form of NETs, vital NETs formation, was proposed.[^25^, ^27^] In detail, Ravindran M et al. have summarized these three different types of vital NETs formation in their review.^[^ ^28^ ^]^

The first evidence pointed to the possibility of NETs roles in cancer was reported in 2012 when neutrophils from tumor‐bearing mice exhibited a strong possibility for producing NETs upon stimulation with lipopolysaccharides (LPS).^[^ ^29^ ^]^ Since then, the research on the bidirectional interplay between cancer and NETs has grown exponentially, becoming the focus for inhibiting tumor growth and metastasis. For instance, in 2013, it was reported that NETs sequestered circulating tumor cells and caused metastasis.^[^ ^30^ ^]^ In 2015, Martha C. Hawes et al. discussed the application of DNase 1 as a clinical tool to prevent or treat cancer and put forward the concept of extracellular DNA of NETs that may function in cancer development.^[^ ^31^ ^]^ From the view of tumor cells on NETs formation, in 2016, metastatic breast cancer cells could prompt neutrophils to come into being metastasis‐supporting NETs.^[^ ^32^ ^]^ In addition, another report indicated that Chi3l1 expressed on triple‐negative breast cancer participated in NETs formation, which led to the decreased T‐cell infiltration.^[^ ^33^ ^]^ In 2023, the relationship of NETs production and hypoxic microenvironment in gastric cancer was demonstrated to augment tumor growth.^[^ ^34^ ^]^ In addition, some complications of tumor also arose from NETs formation. For example, the NETs‐associated thrombosis in cancer patients was also reported.^[^ ^35^ ^]^ The representative historical timeline of NETs functions in tumors is shown in Figure 2.

In the ensuing years, mounting evidence and comprehensive mechanisms for the interactions of NETs and tumors were discovered. For instance, in 2018, it was discovered that dormant cancer cells could be activated by NETs formation during inflammation in mice.^[^ ^36^ ^]^ A milestone in the research process of scientists was Erwei Song and colleagues’ discovery in 2020 that the DNA of NETs promoted cancer metastasis via coiled‐coil domain containing protein 25 (CCDC25).^[^ ^37^ ^]^ In addition, tumor‐associated NETs also produced a more aggressive and tolerogenic tumor microenvironment. As previously reported, the appearance of NETs after surgical procedures plays a pivotal role in the immune microenvironment.^[^ ^38^ ^]^ However, if you search for the reason of NETs formation, there are so many factors. Apart from the tumor‐induced NETs as discussed in the introduction part, NETs formation could be induced by other external factors. One typical example was that bacterial infection also promotes tumor cell metastasis through the formation of NETs. Wu et al. listed some clinical correction of NETs and gut microbiota in CRC progression.^[21a]^ Moreover, some bacteria strains are enriched in patients with CRC, including *Bacteroides fragilis*, *pks* ^*+*^ *Escherichia coli*, *Streptococcus gallolyticus*, and *Morganella morganii*. Furthermore, Shen et al. summarized the main factors that regulate NETs formation during bacterial infection.^[^ ^39^ ^]^ For example, *Staphylococcus aureus* could produce Panton–Valentine leukocidin, which could change neutrophil mitochondria membranes, triggering increased ROS to lead NETs release. Another medium is LPS found in gram‐negative bacteria. On one hand, LPS activates the toll‐like receptor 4 in neutrophils to trigger NETs process.^[^ ^40^ ^]^ On the other hand, NADPH‐oxidase system through Nox receptor binding could also be activated to produce superoxide radicals.^[^ ^41^ ^]^ Recently, chronic stress was reported to increase metastasis via NETs in the microenvironment.^[^ ^42^ ^]^ In summary, the mechanisms for NETs formation are complex.

Beyond the well‐known functions of NETs in tumor metastasis and tumor progression, NETs also contributed to immunosuppression and antitumor immune response.^[^ ^43^ ^]^ In 2021, it was revealed that NETs could suppress T cell responses because of metabolic and functional exhaustion. In addition, treatment with DNase or anti‐PD‐L1 at the time of surgery led to slower tumor growth, which represented a novel and viable method for reshaping tumor immune microenvironment.^[^ ^44^ ^]^ Moreover, a recent study revealed that NETs from patients with pancreatic ductal adenocarcinoma created a microdomain where human arginase 1 (hARG1) could be cleaved by cathepsin S to convert to a higher enzyme activity form. The suppression of T lymphocytes proliferation by NETs‐associated hARG1 could be restored by either using an anti‐hARG1 antibody or preventing cathepsin S cleavage.^[^ ^45^ ^]^ The clinical efficacy of anticancer therapies is markedly limited by tumor therapy resistance. In 2022, Xiang Xia et al. confirmed that NETs in response to infection could promote esophageal cancer cell proliferation, invasion, migration, and epithelial–mesenchymal transition through the transforming growth factor‐β (TGF‐β) pathway.^[14a]^ Moreover, TGF‐β activation was also demonstrated to be one mechanism for chemotherapy resistance, and targeting NETs ameliorates chemotherapy efficacy against lung metastasis.^[^ ^46^ ^]^ For colon cancer, the association of NETs classification and prognosis and response to immunotherapy has been described,^[^ ^47^ ^]^ but formal clinic analyses have been lacking. Similarly, one recent research suggested tumor‐infiltrating NETs as an indicator of clinical outcomes for EWS patients.^[^ ^20^ ^]^ Moreover, the relationship between extracellular DNA and pathological calcification has been receiving increasing attention, but the application is not yet fully developed.^[^ ^48^ ^]^ Although these correlation investigations have not yet been linked to causation, emerging therapeutic strategies aimed at digesting NETs or inhibiting their formation are offering hope for tumor treatment.^[^ ^49^ ^]^

Ongoing clinical trials show that NETs are being used for tumor imaging and therapy. For example, there are five clinical trials registered in ClinicalTrials.gov resource. In two clinical trials, authors aimed to use NETs as new markers for cancer patients with venous thromboembolism (NCT04294589 and NCT03781531). In addition, one clinical trial indicated acetaminophen weakened neoadjuvant chemoimmunotherapy efficacy in patients with non–small cell lung cancer by promoting NETs formation.^[^ ^50^ ^]^

Recently, a bibliometric analysis to help us to understand NETs from 2004 and 2022 was reported.^[^ ^51^ ^]^ In this report, a total of 4866 publications during the period were involved in the bibliometric analysis. The relationship between NETs and cancer is a popular research topic. Therefore, NETs may serve as valuable targets for antitumor therapy.

## 3 Imaging of NETs

Over 20 years of research on NETs have demonstrated that there is a close relationship between NETs and tumors.^[^ ^52^ ^]^ To dynamically visualize the formation process of NETs and evaluate those enzymes’ activity in NETs, some excellent fluorescent probes have been designed and developed,^[^ ^53^ ^]^ and one of them has been used to dynamically evaluate the relationship between NETs and tumors in vivo.^[53c]^

### 3.1 In Vitro Imaging of NETs

The classical imaging technologies (e.g., immunohistochemical staining,^[^ ^54^ ^]^ transmission electron microscope,^[^ ^55^ ^]^ atomic force microscopy,^[^ ^56^ ^]^ and scanning electron microscope) have shown that NETs are a mesh‐like structure composed primarily of DNA, histones, and some enzymes (e.g., elastase, cathepsin G). However, most of them can only provide static results, and the cells there are mainly in the late stage of NETosis. Among them, fluorescence imaging is a noninvasive, simple, and inexpensive process and can be used for dynamic visualization of the formation process of NETs and evaluation the enzymes’ activities when using appropriate fluorescent probes (**Table** **1**).

**Table 1:** Probes for imaging NETs and their components. “↑” shows the enzyme cleavage sites.

Formulation	Imaging groups	Response peptide sequences	Targets	Features	References
hNE–FQ	5‐FAM (fluorophore)	Glu‐Glu‐Ile (EEI)↑‐Nle‐Arg‐Arg‐Lys (RRK)	Neutrophil elastase	Triple‐quenched, tri‐branched FRET probe	[53a]
	Methyl red (quencher)				
H‐NE	Coumarin343 (donor)	Ala‐Pro‐Glu‐Glu‐Ile ↑ Met‐Arg‐Arg‐Gln‐Lys (APEEI↑MRRQK)	Neutrophil elastase	imaging DNA and DNA‐bound neutrophil elastase	[53b]
	5(6)‐TAMRA (acceptor)				
	Hoechst (binding DNA)		DNA	High accurate	
H‐CG	Coumarin343 (donor)	Glu‐Pro‐Phe ↑ Trp‐Glu‐Asp‐Gln‐Lys (EPF↑ WEDQK)	Cathepsin G	Bad performance due to the suppressed activity of Cathepsin G when bound to DNA	[53b]
	5(6)‐TAMRA (acceptor)				
	Hoechst (binding DNA)		DNA		
TNR_1_	Hemicyanine (fluorophore)	Ac‐AAPV↑AAPF↑	Neutrophil elastase, cathepsin G	Tandem response to neutrophil elastase and cathepsin G,	[53c]
				High accurate	
TNR_2_	Hemicyanine (fluorophore)	Ac‐AAPF↑AAPV↑	Neutrophil elastase, cathepsin G	Without tandem response ability. Worse than TNR_1_ in detecting NETosis	[53c]
CDr15	BODIPY	None	Extracellular DNA	High signal‐to‐noise ratio and specificity to DNA in NETs	[53d]
NH_2_–PEG–CdSe (ZnS) Qdots	Qdots	None	NETs	Fluorescence and photoemission electron microscopy (PEEM) and imaging X‐ray photoelectron spectroscopy (XPS) imaging	[58]

**Figure 3:**
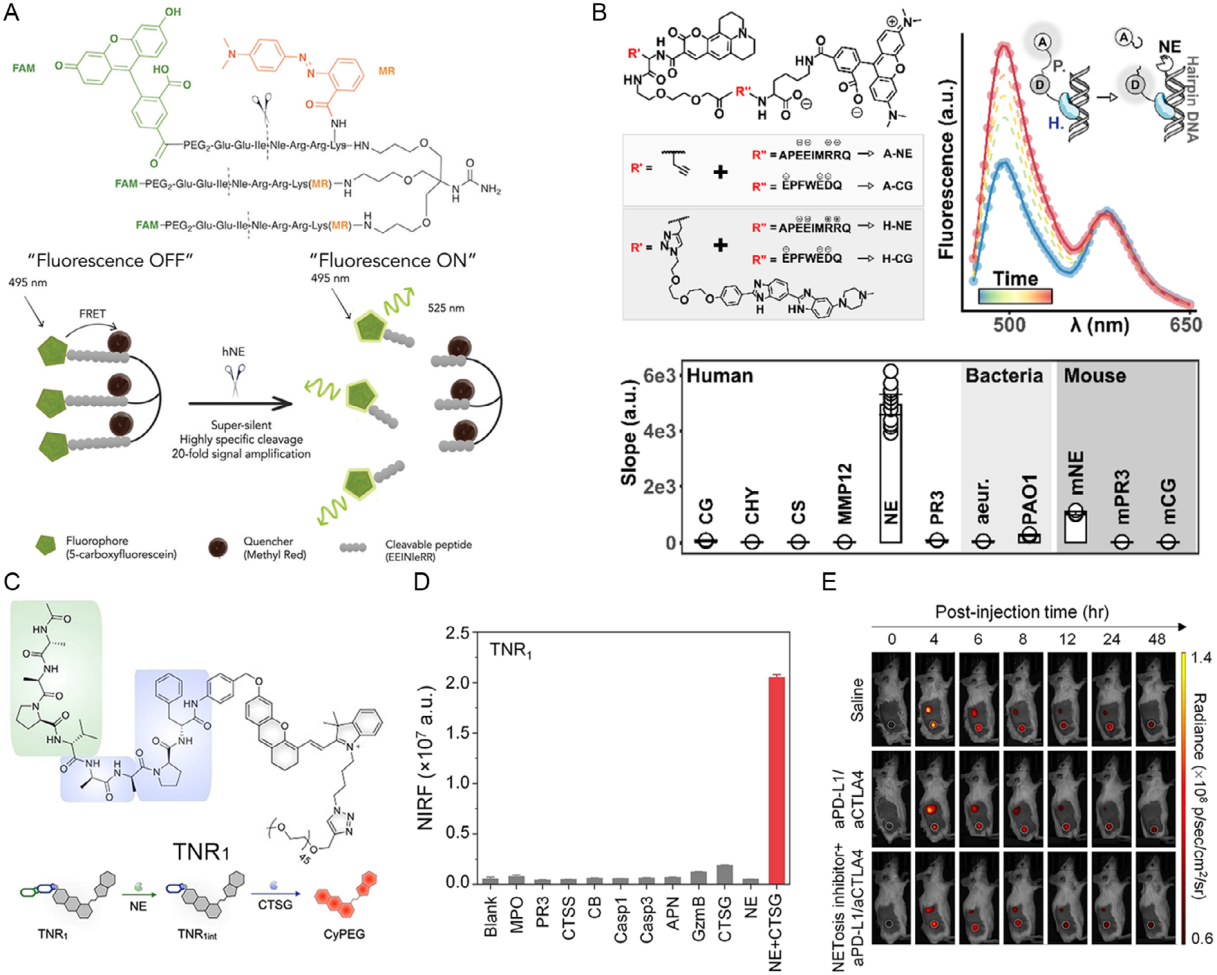
A) Chemical structure and working principle of hNE–FQ for detecting human neutrophil elastase (hNE). Reproduced with permission.^[53a]^ Copyright 2021, Royal Society of Chemistry. B) Chemical structures of different probes, and the performance and good selectivity of H‐NE in response to NE. Reproduced with permission.^[53b]^ Copyright 2020, American Chemical Society. C) Chemical structure of TNR_1_, which can only be lighted up by sequential treatment with NE and cathepsin G (CTSG). D) The good selectivity of TNR_1_ for NE + CTSG. E) The fluorescence signal of TNR_1_ of mice with different treatments. The dotted circles show the position of the tumors. Reproduced with permission.^[53c]^ Copyright 2023, Wiley‐VCH.

In 2021, the group of Mark Bradley reported a triple‐quenched Förster resonance energy transfer (FRET) probe for the sensitive detection of human NE (hNE) in NETs (**Figure** **3**A).^[53a]^ The authors first prepared Fmoc‐Lys(MR)‐OH by coupling quencher methyl red in the side‐chain amino. Then through standard solid‐phase peptide synthesis, hNE–Fluorophore–Quencher (FQ) was obtained by connecting fluorophore 5‐carboxyfluorescein (FAM) in the last coupling step. hNE–FQ has a very low background signal due to the FRET and aggregation‐caused quenching property. When hNE–FQ was cleaved by hNE in the peptide (Glu‐Glu‐Ile [EEI↑]–Nle–Arg‐Arg‐Lys [RRK]), FAM–EEI was released and recovered fluorescence with a 20‐fold enhancement. In the cellular experiment, hNE–FQ can specifically image hNE on extracellular chromatin in PMA pretreated neutrophils.^[53a]^ However, hNE–FQ only detects hNE on NETs, other stains such as DNA dyes (e.g., SYTOX Green) are also needed to synergistically determine whether NETosis has occurred.

To image both DNA and DNA‐bound proteases, the group of Carsten Schultz developed FRET probes H‐NE and H‐CG (cathepsin G [CG]), both containing Hoechst 33 258, Coumarin343 (donor), and 5(6)‐TAMRA (acceptor), to detect NE and CG in NETs, respectively (Figure 3B).^[53b]^ Hoechst 33 258 in the probe allows the molecule to accurately detect DNA‐bound enzymes and can simultaneously image DNA. This study shows that the fluorescence of Hoechst in H‐NE has a good co‐localization with commercial DNA dye (Drap5). At the same time, H‐NE can dynamically evaluate NE activity in donor/acceptor emission ratio channels. H‐CG probe proves that DNA‐bound CG has relatively lower activity than free CG.^[53b]^

Compound of designation red 15 (CDr15), first reported as a dye for staining bacterial extracellular DNA,^[^ ^57^ ^]^ was shown to stain DNA in NETs as well.^[53d]^ The studies of Jong‐Wan Park group showed that CDr15 is superior to STYOX green, due to CDr15 having a higher co‐localization ratio with Cit‐H3, a standard protein used to demonstrate the occurrence of NETs. At the same time, CDr15 can also stain NETs DNA in tumor tissues fixed by 4% paraformaldehyde, which can provide physicians with auxiliary information to assess the status of the tumor of patients.^[53d]^

Except for the aforementioned dyes that specifically bind or detect some component of NETs, some nanoparticles without obvious targeting were also reported can stain NETs. The group of K. Uvdal observed the formation of NETs in neutrophils activated by NH_2_–polyethylene glycol (PEG)–CdSe (ZnS) Qdots by using photoemission electron microscopy and imaging X‐ray photoelectron spectroscopy.^[^ ^58^ ^]^ Moreover, some new techniques (i.e., expansion microscopy^[^ ^59^ ^]^and microfluidics^[^ ^60^ ^]^) are also used for NETs detection. In addition, some simplified experiments using safranin^[^ ^61^ ^]^ and smear assay^[^ ^62^ ^]^ are proposed to quickly visualize NETs at a lower cost. Recently, a label‐free refractive‐index‐based 3D tomographic imaging was reported to achieve 3D imaging of cellular structures.^[^ ^63^ ^]^ Therefore, by using this technology, the NETs process could be observed with high accuracy. The attempt in utilizing artificial intelligence (AI) tools for the investigation of cancer cytopathology has been proposed and developed.^[^ ^64^ ^]^ To our knowledge, the investigation of obtaining more data about NETs using AI has not been done before. In the future, the use of AI may help us to understand the NETs in detail.

### 3.2 In Vivo Imaging of NETs

Compared to in vitro imaging, the dynamic observation of NETs or NETosis in vivo requires additional consideration of biosafety. In 2023, the group of Pu reported a PEG‐modified tandem‐locked NETosis reporter 1 (TNR1) probe, which contains an octapeptide (Ac‐Ala‐Ala‐Pro‐Val (AAPV)↑Ala‐Ala‐Pro‐Phe (AAPF)↑) to monitor NE and cathepsin G (CTSG) simultaneously.^[53c]^ Without cleaving AAPV sequence by NE first, the residue V will hinder CTSG from binding to the probe, which is still non‐fluorescent. Therefore, TNR1 can only be light‐up by responding to NE and CTSG sequentially (Figure 3C,D). This tandem response property makes TNR1 hold high accuracy in identifying neutrophils with NETosis from normal activated neutrophils, which also express NE. The control probe TNR2, containing Ac‐AAPF↑AAPV↑, can be directly activated by NE, so its accuracy in detecting NETosis is weaker than TNR1. The near‐infrared emission and fluorescence turn‐on properties of the probe allow for in vivo detection of NETosis. In addition, TNR1 also successfully demonstrates the negative correlation between NETosis and cancer immunotherapeutic efficacy by detecting the fluorescence signal (Figure 3E).^[53c]^

## 4 Pharmacological Modulation of NETs for Tumor Therapy

### 4.1 Destruction of NETs Components to Inhibit Tumor Therapy

DNA is the main skeleton of NETs. Physiologically, the DNA of NETs is degraded by serum DNases such as DNase‐1, which is an endonuclease and is distributed in plasma.^[^ ^65^ ^]^ DNases are derived from non‐hematopoietic cells, which mainly digested DNA strands in the absence of protein. Moreover, it is widely used as a molecular biology tool. To date, one kind of DNase‐1, human recombinant DNase‐1 (Pulmozyme), has been approved by the U.S. Food and Drug Administration (FDA) in 1993 and is used for cystic fibrosis to digest extracellular DNA fibers, which was scattered among the lungs.^[^ ^66^ ^]^ Interestingly, over 50 years of progress have also shown the effectiveness of DNase‐1 in improving cancer therapy.^[^ ^31^ ^]^ Recently, Tsung et al. reported that adeno‐associated virus‐mediated DNase‐1 liver gene transfer following a single intravenous injection suppressed the development of liver metastases in a mouse model of CRC liver metastasis.^[^ ^67^ ^]^

DNase‐1 preferentially digests naked double‐stranded DNA (dsDNA), whereas chromatin is the substrate for DNase1L3. For example, defective DNase1L3 aggravates NETs DNA‐triggered hepatocellular carcinoma invasion in diabetic conditions via cyclic guanosine monophosphate (GMP)–adenosine monophosphate (AMP) synthase (cGAS) and the noncanonical nuclear factor kappa‐B (NF‐κB) pathway.^[^ ^68^ ^]^ Moreover, DNase1L3 was downregulated in human tumors, which is one reason for poor patient survival.^[^ ^69^ ^]^ Therefore, the combination of DNase‐1 and DNase1L3 could afford efficient NETs degradation.^[^ ^70^ ^]^ For patients with lower DNase levels, DNase treatment may be especially helpful. Moreover, more attention should be paid on the degradation product of NETs. For example, one earlier work has indicated that NETs could be converted to deoxyadenosine with the help of *Staphylococcus aureus* while deoxyadenosine can induce the caspase‐3‐mediated death of immune cells.^[^ ^71^ ^]^ Therefore, the balance between NETs formation and degradation should be not ignored.

In summary, the DNase that cleaves single‐stranded and dsDNA has shown potential for disintegration of NETs in cancer patients. However, considering the low serum stability and fast deactivation, how to improve its enzyme activity and extend blood circulation should be considered for cancer treatment.

### 4.2 Inhibition of NETs Formation to Enhance Tumor Therapy

Peptidyl arginine deiminase 4 (PAD4), a neutrophil‐enriched nuclear enzyme, is involved in the formation of NETs by catalyzing histone hypercitrullination in a Ca^2+^‐dependent manner, which in turn leads to chromatin decondensation.^[^ ^72^ ^]^ In regard to inhibition PAD4, several PAD4 inhibitors (e.g., GSK484, YW4‐03, JBI‐589, and BMS‐P5) have the potential to inhibit PAD4, leading to decreased NETs (**Table** **2**). Oftentimes, the PAD inhibitor application was demonstrated to hold a superior antitumor effect. As an example, GSK484 improved the radiosensitivity of CRC cells and induced cell death by promoting dsDNA breaks.^[^ ^73^ ^]^

**Table 2:** Summary of drugs whose mechanisms of action may involve inhibition of NETs formation for tumor therapy.

Drug name	Mechanisms of action	Cancer therapy application	References
GSK484	Pharmacological targeting of peptidylarginine deiminase 4 (PAD4) and its inhibitor	Breast cancer	[73, 106]
YW4‐03	The PAD4 inhibitor	Breast cancer	[107]
JBI‐589	Reduced CXCR2 expression and blocked neutrophil chemotaxis	Lung cancer	[108]
BMS‐P5	The PAD4 inhibitor	Multiple myeloma	[109]
Protectin D1	PD1 ameliorates acute pancreatitis by decreasing early infiltration of neutrophils into the pancreas and NETs formation through PAD4.	Pancreatic cancer	[110]
Sivelestat sodium	A specific NE inhibitor, and it could inhibit postoperative systemic inflammatory reactions after radical surgery for esophageal cancer.	Esophageal cancer and gastric carcinoma	[111]
Ivermectin	Suppression of GSDMD oligomerization, and the GSDMD‐dependent NETs formation is blocked.	Melanoma cancer	[80]
Dihydrotanshinone I	Suppression of the NETs formation by restraining tissue inhibitor of matrix metalloproteinase‐1 expression	Breast cancer	[112]
Thrombomodulin	The HMGB1 of NETs may lead to aggravate the malignancy of cancer cells, and thrombomodulin could degrade HMGB1.	Pancreatic cancer	[77]
Histidine‐rich glycoprotein (HRG)	The reduced IL‐8 level was observed because of PI3K and NF‐κB inactivation, thereby decreasing neutrophil recruitment. In addition, IL8–MAPK and NF‐κB pathway activation was inhibited, leading to decreased NETs formation.	Liver cancer	[78]
Kaempferol	Kaempferol decreases ROS production in neutrophils through NADPH/ROS–NETs signaling.	Breast cancer	[113]

NE is one kind of serine proteases normally located in polymorphonuclear neutrophils. During the NETs formation, the NE decorated on the DNA could have its proteolytic activity for a long time. NE has been demonstrated to have a pro‐tumoral role in breast, lung, prostate, and colon cancer.^[^ ^74^ ^]^ Of note, clinically approved sivelestat sodium, an NE inhibitor, was also shown to be beneficial after surgical resection of esophageal cancer.^[^ ^75^ ^]^ However, another research showed that NE selectively killed cancer cells and attenuated tumorigenesis.^[^ ^76^ ^]^ Such opposing results on pro‐tumoral/antitumoral roles of NE suggest the involvement of other associated parameters that collectively dictate the effect of NETs. Therefore, there is a need to thoroughly investigate its function for archiving good anticancer therapy.

In addition to inhibiting NETs formation by small molecule inhibitors, some biomacromolecules (e.g., thrombomodulin‐ and histidine‐rich glycoprotein [HRG]) have also been shown to inhibit NETs formation for improving tumor therapy. Fujiwara et al. demonstrated that high‐mobility group box 1 (HMGB1) originating from NETs could aggravate the malignancy of cancer cells. Subsequently, they used thrombomodulin to degrade HMGB1 to inhibit the production of NETs, thereby inhibiting pancreatic cancer metastasis to the liver.^[^ ^77^ ^]^ In addition, one research indicated HRG, a secretory glycoprotein, bound to FCγR1 on the neutrophil membrane while inhibiting PI3K and NF‐κB activation, thereby reducing interleukin‐8 (IL‐8) secretion to decrease neutrophil recruitment. In addition, the HRG effect in reduced NETs formation was demonstrated to be linked with the inhibition of IL8–mitogen‐activated protein kinase (MAPK) and NF‐κB pathway activation and ROS production.^[^ ^78^ ^]^

NET formation was also reported to be associated with pyroptosis, which was another form of nonapoptotic cell death.^[^ ^79^ ^]^ Ye et al. studied many compounds for inhibiting NETs and found that ivermectin could abrogate NETs.^[^ ^80^ ^]^ Moreover, the mechanism proposed by them was that it could target a pyroptotic driving factor gasdermin D (*Gsdmd*), and showed a *K*_d_ of 267.96 nM by microscale thermophoresis assay. However, another group showed that NET formation is GSDMD‐independent because *Gsdmd*‐deficient mouse neutrophils could also produce NETs compared to the wide‐type mouse.^[^ ^81^ ^]^ These findings showed the complexity behind the role of GSDMD during the NETs process. A further understanding of its mechanisms will be helpful for the precise classification of NETs and guiding cancer therapy.

Tailoring drugs for modulation of NETs fate was important. Especially, Chiang et al. reported resolvin T‐series could reduce NETs. One major mechanism was that they could stimulate NETs clearance by mouse macrophages through protein kinase A and AMP‐activated protein kinase inhibition.^[^ ^82^ ^]^

To sum up, we have summarized the reported agents utilized for the inhibition of NETs formation and their antitumor applications in this section, as shown in Table 2. Each of these methods has its inherent advantages and disadvantages depending on the specific application conditions. Moreover, some other agents (e.g., H_2_, albumin, and chloroquine) were also demonstrated that they could inhibit the formation of NETs.^[^ ^83^ ^]^ Although these agents are promising in preclinical studies, their effect on enhancing tumor therapy remains to be tested in clinical studies.

### 4.3 Usage of Nanoparticles (NPs) to Affect NETs for Tumor Therapy

Holding the aforementioned promising drugs in hand, which can act on different molecular targets to modulate NETs and thus exert antitumor effects, the following task is how to deliver therapeutic drugs to NETs in an effective and selective way. Nano‐based drug delivery systems exhibit great advantages due to their rich morphology, modifiability, and stimulus responsiveness. These delivery systems can also improve the behavior of drugs in vivo, enhance their drug stability, and acquire better‐targeted drug delivery to NETs through rational design.

**Figure 4:**
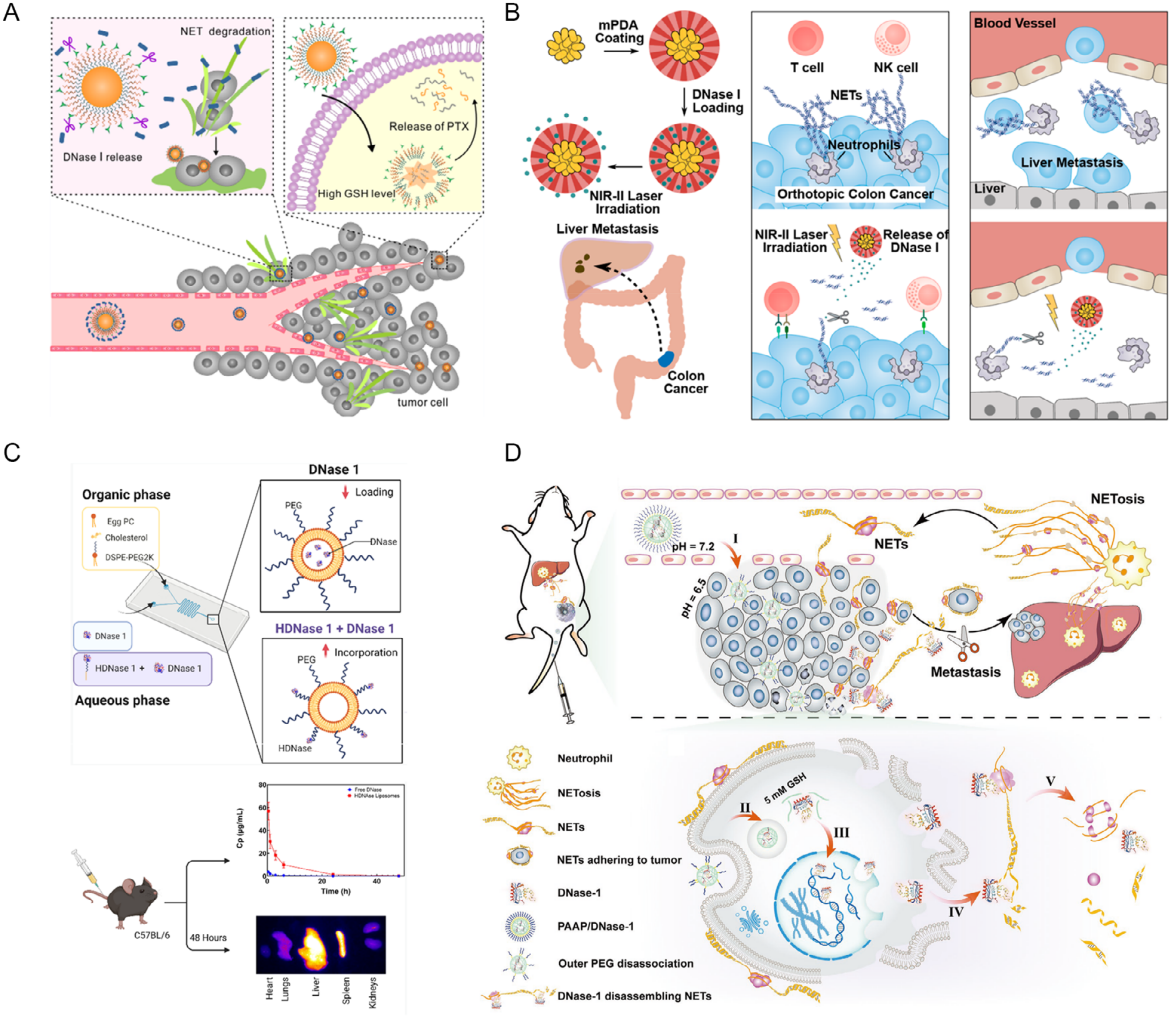
DNase‐1‐loaded nanoparticles for destruction of NETs to improve tumor therapy efficiency. A) The mP–NPs–DNase/PTX with the function of regulation of NETs can inhibit malignant tumor growth and distant metastasis. Reproduced with permission.^[^ ^84^ ^]^ Copyright 2021, American Chemical Society. B) Schematic illustration for the construction and NIR‐II‐responsive DNase‐1‐loaded nanosystem, which could mediate NETs destruction for improving anticancer efficacy of immunotherapy and inhibiting liver metastasis. Reproduced with permission.^[^ ^85^ ^]^ Copyright 2022, American Chemical Society. C) DNase‐1‐loaded liposomes have a PK profile compared to the free enzyme. Reproduced with permission.^[^ ^87^ ^]^ Copyright 2022, American Chemical Society. D) The schematic of the working principle of PAAP/DNase‐1 nanoparticles. Reproduced with permission.^[^ ^88^ ^]^ Copyright 2021, Wiley‐VCH Verlag GmbH & Co. KGaA, Weinheim.

A general strategy for increasing drugs’ therapeutic potential of a drug involves connecting it to special nanoparticles. If used with a drug delivery system, this method has the potential to maintain anticancer drug delivery and therefore enhance efficacy. As shown in **Figure** **4**A, Yin et al. constructed a kind of mP–NPs–DNase/paclitaxel (PTX) to destroy tumor‐associated NETs, which incorporates a PTX prodrug nanoparticle core and a composite shell containing DNase‐1.^[^ ^84^ ^]^ On one hand, this delivery technology utilized the higher MMP‐9 level of NETs to release DNase‐1 since poly‐L‐DNase‐1 was conjugated with lysine through the MMP‐9‐cleavable Tat‐peptide. On the other hand, the high level of reduced glutathione within tumor cells was also used to release PTX to kill tumor cells. They used this nanocarrier to prevent the 4T1 tumor metastasis to the lung. The survival curve demonstrated that this system achieved significant achievements in prolonging the median survival time of mice to 32 days while that for the control group was only 25 days. However, when chemical modification of DNase‐1 was carried out, its activity should be of more significant concern.

Instead of modifying DNase‐1 directly on peptide beads for NETs destruction, several other examples of loading DNase on nanoparticles exist, including the use of chemical modification for enhancing circulation time and stability. For instance, a nanosystem coated with a mesoporous polydopamine shell was used to deliver DNase due to the presence of the surface mesoporous structure (Figure 4B). The controlled release of DNase‐1 with the help of near‐infrared‐II (NIR‐II) light irradiation removed the extracellular NETs in both orthotopic CRC and liver, resulting in improved immune checkpoint therapy of CRC and decreased liver metastasis.^[^ ^85^ ^]^ Of course, our group also constructed an nanosystem loading with DNase‐1 and doxorubicin for tumor therapy.^[^ ^86^ ^]^ Moreover, one group explored the possibility of liposomal nanocarriers to improve the pharmacokinetic (PK) profile of DNase‐1.^[^ ^87^ ^]^ First, aliphatic polymer C_18_–PEG_4_–N‐hydroxysuccinimide (NHS) was conjugated with DNase‐1, forming a modified hydrophobic form of DNase‐1. Then, the modified hydrophobic form of DNase‐1 was anchored to the surface of a PEGylated liposome. In vitro endonuclease activity showed that no significant difference in activity was observed between DNase‐loaded liposomes and native DNase‐1. When they are injected intravenously, the half‐life of DNase‐1 delivered by this kind of liposome is increased by threefold (Figure 4C).

Another strategy was to use DNase‐1 entrapped nanoparticles for degrading NETs (Figure 4D). Zhu et al. used polyamino acid‐polyethylene glycol (PAAP) for loading DNase‐1. The results showed that PAAP/DNase‐1 could degrade chromatin to induce apoptosis, accompanied by cell membrane rupture. More importantly, the released DNase‐1 can disassemble NET–DNA to inhibit liver metastasis due to intravenous injection of NETs. In all, PAAP/DNase‐1 treatment enhanced the efficiency of inhibiting tumor growth by disassembling the NET–DNA.^[^ ^88^ ^]^ Because the NETs exist within both the tumor tissue and circulation system, nanocarriers loading with DNase‐1 can be used as a general strategy for the degradation of NETs.

Different from the destruction of NETs by DNase‐1‐loaded nanoparticles, another research attempted to use a novel PAD4 inhibitor ZD‐E‐1M, which could self‐assemble into nanodrug ZD‐E‐1.^[^ ^89^ ^]^ In vivo, ZD‐E‐1 exhibited great potential in inhibiting tumor growth and metastasis by suppressing PAD4 activity and NETs formation. Moreover, it could increase immune cells in tumor‐bearing mice.

**Figure 5:**
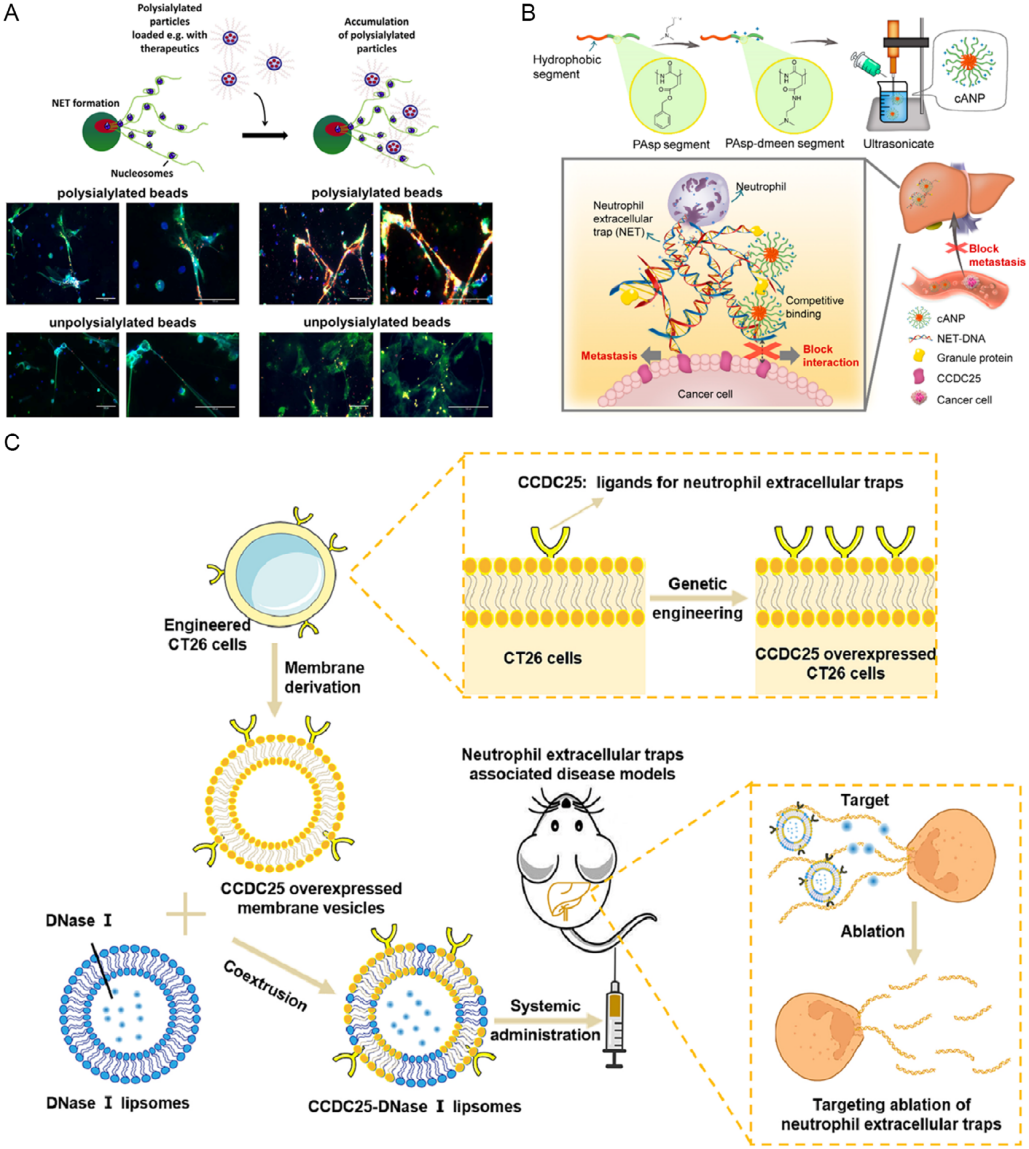
Targeted delivery with nanomaterials for NETs with targeting abilities. A) The concept illustration of polysialylated nanoparticles accumulation on NETs. NETs were visualized by 4′,6‐diamidino‐2‐phenylindole (DAPI) and an antibody against NE. Scale bar: 100 μm. Reproduced under the terms of the CC‐BY license.^[^ ^90^ ^]^ Copyright 2017, The Author(s). Published by Frontiers Media SA. B) Schematic illustration of cANP construction and mechanism of using cationic polymers to bind with NET–DNA for the sake of inhibiting NET‐involved cancer distant metastases to the liver. Reproduced with permission.^[^ ^91^ ^]^ Copyright 2023, American Chemical Society. C) Schematic illustration of DNase‐1‐loaded hybrid liposomes merged with CCDC25 expressed CT26 cell membrane for targeted degradation of NETs. Reproduced with permission.^[^ ^95^ ^]^ Copyright 2023, Elsevier.

For the usage of nanoparticles to affect NETs for tumor therapy, another challenge is how to improve the NETs‐targeted delivery. Inspired by that, sialidases are frequently located in areas of NETs formation, Galuska et al. oxidized the terminal sialic acid residue in the nonreducing state and connected it to amino‐modified particles.^[^ ^90^ ^]^ They expected that these polysialylated nanoparticles could accumulate on NETs (**Figure** **5**A). Their results showed that polysialylated fluorescence beads could abundantly accumulate on NETs. About 97% of the red fluorescence signals were overlapped on NETs in two concentrations of polysialylated fluorescence beads. In comparison, unpolysialylated ones showed less binding to NETs. Further investigation demonstrated that polysialic acid chains could only bind histones rather than the DNA.

Using a different principle, Liang et al. reported nanoparticulate cationic poly(amino acids)s could block cancer metastases by eliminating NETs (Figure 5B).^[^ ^91^ ^]^ They first prepared poly(aspartic acid) (PAsp)‐based cationic nanoparticle (cANP), which was constructed by self‐assembly of the amphiphilic block copolymer of PAsp with 100% dmeem modification. They first demonstrated that these cationic polymers affected the interaction between NET–DNA and CCDC25. Moreover, both PAsp‐100% and cANP‐100% reduced MDA‐MB‐231 cell adhesion to the plates coated with NET–DNA. At last, they presented that the nanoparticulate material may be promising in antimetastatic efficacy. The use of self‐assembling peptide nanomaterials for protein therapeutics might be a viable choice.

To further improve the targeting performance, another report by Stavrou and co‐workers explored the application of NETs‐targeting peptides on the nanoparticles for targeting activated neutrophils.^[^ ^92^ ^]^ The key principle was that the NE outcompetes the α1‐antitrypsin (AAT).^[^ ^93^ ^]^ Considering that the NE was abundant on NETs, an NE‐binding peptide (NEBP) with the amino acid residue order of CGEAIPMSIPPEVK was designed, which was derived from the reactive loop of AAT. The following NEBP–NPs mainly bound to activated neutrophils and NETs instead of inactive cells, whereas these NPs without NEBP did not colocalize with either inactive or activated neutrophils. Therefore, NEBP‐modified nanoparticles were promising for NETs destruction for tumor therapy. In addition, to prepare a nanopreparation that can specifically recognize and destroy NETs, Filipczak et al. reported that 2C5 antibody‐modified micelles coated with DNase‐1 have better properties in recognizing the NETs and promoting their degradation.^[^ ^94^ ^]^ In a similar but distinct example (Figure 5C), Wang et al. constructed a special cell membrane‐derived liposomes loaded with DNase‐1 for disintegrating NETs. Interestingly, the specific interaction between CCDC25 and NETs was referred to construct biomimetic hybrid liposomes capable of targeting NETs, in which CCDC25‐overexpressing tumor cell membrane was used. Their results showed that this kind of liposome could inhibit CRC liver metastases.^[^ ^95^ ^]^

Apart from the aforementioned nanoparticles involving the DNase application, some specific nanoparticles could also digest DNA with DNase‐like activity. Tang et al. found that aggregation‐induced emission artificial enzymes have enduring DNase‐mimetic activity.^[^ ^96^ ^]^ Of course, the role of NETs in tumor development is not absolute. Recently, one research indicated that chemotherapy‐induced NETs could kill cancer cells.^[^ ^97^ ^]^ Therefore, it is of great significance to investigate the differences of NETs, including the structure, components, and roles. In addition, it has to be kept in mind that some kinds of nanoparticles or biomaterials are found to induce NETosis.^[^ ^98^ ^]^ Therefore, the candidate nanoparticles in the future have to be evaluated for their capacity to produce NETosis before further in vivo application.

## 5 Conclusion and Outlook

Imaging and therapy of tumor‐associated NETs offer an alternative way for cancer therapy and are increasingly growing in scope. With more understanding of the bidirectional interplay between cancer and NETs, various strategies for the modulation of NETs are developed for therapeutic applications. However, if NET molecules were used as therapeutic targets in oncology, it will require dedicated effort on both the fundamental and applied levels. We see the following areas as being particularly worthy of study.

### 5.1 Exploring the Bidirectional Relationship between NETs and Tumors in Depth

First, for in vitro NETs formation, the characteristics of NETs may vary with stimulus type. One example is the NET chromatin (NETchr), which could be obtained by high‐speed centrifugation after NETs formation.^[^ ^99^ ^]^ Moreover, Triton X‐100 was also be used to disrupt the integrity of cell membrane for entire NETs release.^[^ ^100^ ^]^ Recently, DNA outflow but not NETs formation was suggested if PMA stimulation was carried out under hypoxia.^[^ ^101^ ^]^ Although “NETosis” was also used in many reports, the Nomenclature Committee on Cell Death in 2018 still recommends that the term “NETosis” should be avoided, in the absence of evidence of cell death.^[^ ^28^ ^]^ As discussed previously, the diverse and sometimes contradictory roles of NETs have been discovered. Therefore, it is important to investigate the NETs formation and features in depth. Second, the bidirectional relationship between NETs and tumors should also be further studied. On one hand, it is increasingly believed that they operate via a complex mechanism. On the other hand, the role of NETs in tumor development may be attributed to the differences in tumor categories, tumor model, and immune condition of the host. Likely, an optimal design concept and careful consideration of the competing beneficial and detrimental effects will prove necessary if the benefits of NETs are to be fully realized.

### 5.2 Developing NETs‐Derived Signature to Predict the Prognosis of Some Specific Tumors

Although formal clinic analyses have been lacking, the association of NETs classification and prognosis and response to immunotherapy has been described for colon cancer.^[^ ^47^ ^]^ Recently, one group also described the prognostic value of NETs‐derived signature (NETScore) in osteosarcoma.^[^ ^102^ ^]^ Thus, it is of significance for developing NETs‐derived signature to predict the prognosis of some specific tumors. Of note, various tumor markers, such as secreted proteins and carcinoembryonic antigen, may coexist during NETs formation. Moreover, these makers are not fixed but can be interchanged. Therefore, developing probes for imaging or detection of NETs with more accuracy and sensibility may have a higher clinical value. Future applications may benefit from detecting more than one marker at a time, which may possibly provide a more precise index for predicting the prognosis of specific tumors than is possible by the use of single biomarker.

### 5.3 Optimal Preparations to Regulation NETs for Suitable Clinical Translation

The most pressing need related to therapy of tumors based on NETs in our view is to determine which cancer morphologies would benefit most from its application. In the future, we envision the continued use of pharmacological modulation of tumor‐associated NETs for improving tumor therapy efficiency. We also envision the continued growth in emerging agents that digest NETs or inhibit NETs formation without affecting others. In this regard, further advances in tumor‐associated NETs will be instrumental in developing agents that exhibit desirable properties across the board. Moreover, an efficacious combination therapy as well as optimal preparations may help to achieve safe and good therapeutic effect. According to a previous study, combination of tumor acidity neutralizer and NETs lyase with one hydrogel improved natural killer cells to inhibit postsurgical hepatocellular carcinoma recurrence without systemic toxicity.^[^ ^103^ ^]^ Zhou et al. prepared another hydrogel for co‐deliver DNase‐1‐encapsulated poly(lactic‐co‐glycolic acid) (PLGA) nanoparticles and an unselective β‐adrenergic receptor blocker against cancer recurrence and metastasis.^[^ ^104^ ^]^ Apart from hydrogel application, self‐assembled nanosytems by using DNase‐1 and other therapeutic agents may also be promising. In addition, probiotic therapy may open up the possibility of replacement of DNase‐1 because it could also inhibit NETs.^[^ ^105^ ^]^ Importantly, they should be further assessed on large animal models, such as macaques or dogs, especially for understanding whether their diagnostic and therapeutic capabilities are still good. Taken together, these advances will fuel the sustained evolution of NETs in their roles as essential tools in antitumor medicine.

## Conflict of Interest

The authors declare no conflict of interest.

## References

[1] Z. Li , J. Zou , X. Chen , Adv. Mater. 2023, 35, 2209529. 10.1002/adma.202209529 36445169

[2] E. A. Sweet‐Cordero , J. A. Biegel , Science 2019, 363, 1170. 30872516 10.1126/science.aaw3535 PMC7757338

[3] T. N. Mayadas , X. Cullere , C. A. Lowell , Annu. Rev. Pathol. 2014, 9, 181. 24050624 10.1146/annurev-pathol-020712-164023 PMC4277181

[4] a) D. C. Doeing , J. L. Borowicz , E. T. Crockett , BMC Clin. Pathol. 2003, 3, 3; 12971830 10.1186/1472-6890-3-3 PMC201031

[5] E. Kolaczkowska , P. Kubes , Nat. Rev. Immunol. 2013, 13, 159. 23435331 10.1038/nri3399

[6] a) S. Basu , G. Hodgson , M. Katz , A. R. Dunn , Blood 2002, 100, 854; 12130495 10.1182/blood.v100.3.854

[7] a) V. Brinkmann , U. Reichard , C. Goosmann , B. Fauler , Y. Uhlemann , D. S. Weiss , Y. Weinrauch , A. Zychlinsky , Science 2004, 303, 1532; 15001782 10.1126/science.1092385

[8] T. Yang , J. Yu , T. Ahmed , K. Nguyen , F. Nie , R. Zan , Z. Li , P. Han , H. Shen , X. Zhang , S. Takayama , Y. Song , Sci. Adv. 2023, 9, eadf2445. 37115934 10.1126/sciadv.adf2445 PMC10146876

[9] V. Mutua , L. J. Gershwin , Clin. Rev. Allergy Immunol. 2021, 61, 194. 32740860 10.1007/s12016-020-08804-7 PMC7395212

[10] J. M. Adrover , S. A. C. McDowell , X.‐Y. He , D. F. Quail , M. Egeblad , Cancer Cell 2023, 41, 505. 36827980 10.1016/j.ccell.2023.02.001 PMC10280682

[11] S. Berger‐Achituv , V. Brinkmann , U. A. Abed , L. I. Kühn , J. Ben‐Ezra , R. Elhasid , A. Zychlinsky , Front. Immunol. 2013, 4, 48. 23508552 10.3389/fimmu.2013.00048 PMC3589747

[12] M. M. Rivera‐Franco , E. Leon‐Rodriguez , J. J. Torres‐Ruiz , D. Gómez‐Martín , E. Angles‐Cano , S.‐G. M. de la Luz , Pathol. Oncol. Res. 2020, 26, 1781. 31656990 10.1007/s12253-019-00763-5

[13] L.‐M. Mauracher , L. Hell , F. Moik , M. Krall , C. Englisch , J. Roiß , E. Grilz , T. M. Hofbauer , C. Brostjan , S. Knapp , C. Ay , I. Pabinger , Res. Pract. Thromb. Haemostasis 2023, 7, 100126. 37063752 10.1016/j.rpth.2023.100126 PMC10099311

[14] a) X. Xia , Z. Zhang , C. Zhu , B. Ni , S. Wang , S. Yang , F. Yu , E. Zhao , Q. Li , G. Zhao , Nat. Commun. 2022, 13, 1017; 35197446 10.1038/s41467-022-28492-5 PMC8866499

[15] a) A. M. Stehr , G. Wang , R. Demmler , M. P. Stemmler , J. Krug , P. Tripal , B. Schmid , C. I. Geppert , A. Hartmann , L. E. Muñoz , J. Schoen , S. Völkl , S. Merkel , C. Becker , G. Schett , R. Grützmann , E. Naschberger , M. Herrmann , M. Stürzl , J. Pathol. 2022, 256, 455; 34939675 10.1002/path.5860

[16] Q. Li , W. Chen , Q. Li , J. Mao , X. Chen , Front. Immunol. 2022, 13, 1019967. 36225931 10.3389/fimmu.2022.1019967 PMC9549764

[17] J. Deng , Y. Kang , C.‐C. Cheng , X. Li , B. Dai , M. H. Katz , T. Men , M. P. Kim , E. A. Koay , H. Huang , R. A. Brekken , J. B. Fleming , JCI Insight 2021, 6, e146133. 34237033 10.1172/jci.insight.146133 PMC8492346

[18] R. Herranz , J. Oto , M. Hueso , E. Plana , F. Cana , M. Castaño , L. Cordón , D. Ramos‐Soler , S. Bonanad , C. D. Vera‐Donoso , M. Martínez‐Sarmiento , P. Medina , Front. Immunol. 2023, 14, 1171065. 37275882 10.3389/fimmu.2023.1171065 PMC10237292

[19] S. Tomás‐Pérez , J. Oto , C. Aghababyan , R. Herranz , A. Cuadros‐Lozano , E. González‐Cantó , C. B. Mc , J. Arrés , M. Castaño , F. Cana , L. Martínez‐Fernández , N. Santonja , R. Ramírez , A. Herreros‐Pomares , S. Cañete‐Mota , A. Llueca , J. Marí‐Alexandre , P. Medina , J. Gilabert‐Estellés , Front. Immunol. 2023, 14, 1111344. 36817483 10.3389/fimmu.2023.1111344 PMC9936152

[20] R. Shukrun , S. Baron , V. Fidel , A. Shusterman , O. Sher , N. Kollender , D. Levin , Y. Peled , Y. Gortzak , Y. Ben‐Shahar , R. Caspi , S. Gordon , M. Manisterski , R. Elhasid , Cancer Sci. 2024, 115, 36. 37915266 10.1111/cas.15992 PMC10823276

[21] a) J. Wu , W. Dong , Y. Pan , J. Wang , M. Wu , Y. Yu , Front. Immunol. 2023, 14, 1296783; 37936694 10.3389/fimmu.2023.1296783 PMC10626548

[22] a) Á. Teijeira , S. Garasa , M. Gato , C. Alfaro , I. Migueliz , A. Cirella , C. de Andrea , M. C. Ochoa , I. Otano , I. Etxeberria , M. P. Andueza , C. P. Nieto , L. Resano , A. Azpilikueta , M. Allegretti , M. de Pizzol , M. Ponz‐Sarvisé , A. Rouzaut , M. F. Sanmamed , K. Schalper , M. Carleton , M. Mellado , M. E. Rodriguez‐Ruiz , P. Berraondo , J. L. Perez‐Gracia , I. Melero , Immunity 2020, 52, 856; 32289253 10.1016/j.immuni.2020.03.001

[23] X. Su , A. Brassard , A. Bartolomucci , I. Dhoparee‐Doomah , Q. Qiu , T. Tsering , R. Rohanizadeh , O. Koufos , B. Giannias , F. Bourdeau , L. Feng , J. Messina‐Pacheco , S. Leo , V. Sangwan , D. Quail , J. Tankel , J. Spicer , J. V. Burnier , S. D. Bailey , L. Ferri , J. Cools‐Lartigue , J. Extracell. Vesicles 2023, 12, 12341. 37563798 10.1002/jev2.12341 PMC10415595

[24] H. Takei , A. Araki , H. Watanabe , A. Ichinose , F. Sendo , J. Leukocyte Biol. 1996, 59, 229. 8603995 10.1002/jlb.59.2.229

[25] a) T. A. Fuchs , U. Abed , C. Goosmann , R. Hurwitz , I. Schulze , V. Wahn , Y. Weinrauch , V. Brinkmann , A. Zychlinsky , J. Cell Biol. 2007, 176, 231; 17210947 10.1083/jcb.200606027 PMC2063942

[26] D. N. Douda , M. A. Khan , H. Grasemann , N. Palaniyar , Proc. Natl. Acad. Sci. U. S. A. 2015, 112, 2817. 25730848 10.1073/pnas.1414055112 PMC4352781

[27] B. G. Yipp , B. Petri , D. Salina , C. N. Jenne , B. N. Scott , L. D. Zbytnuik , K. Pittman , M. Asaduzzaman , K. Wu , H. C. Meijndert , S. E. Malawista , C. A. de Boisfleury , K. Zhang , J. Conly , P. Kubes , Nat. Med. 2012, 18, 1386. 22922410 10.1038/nm.2847 PMC4529131

[28] M. Ravindran , M. A. Khan , N. Palaniyar , Biomolecules 2019, 9, 365. 31416173 10.3390/biom9080365 PMC6722781

[29] M. Demers , D. S. Krause , D. Schatzberg , K. Martinod , J. R. Voorhees , T. A. Fuchs , D. T. Scadden , D. D. Wagner , Proc. Natl. Acad. Sci. U. S. A. 2012, 109, 13076. 22826226 10.1073/pnas.1200419109 PMC3420209

[30] J. Cools‐Lartigue , J. Spicer , B. McDonald , S. Gowing , S. Chow , B. Giannias , F. Bourdeau , P. Kubes , L. Ferri , J. Clin. Invest. 2013, 123, 3446. 23863628 10.1172/JCI67484 PMC3726160

[31] M. C. Hawes , F. Wen , E. Elquza , Cancer Res. 2015, 75, 4260. 26392072 10.1158/0008-5472.CAN-15-1546

[32] J. Park , R. W. Wysocki , Z. Amoozgar , L. Maiorino , M. R. Fein , J. Jorns , A. F. Schott , Y. Kinugasa‐Katayama , Y. Lee , N. H. Won , E. S. Nakasone , S. A. Hearn , V. Kuttner , J. Qiu , A. S. Almeida , N. Perurena , K. Kessenbrock , M. S. Goldberg , M. Egeblad , Sci. Transl. Med. 2016, 8, 361ra138. 10.1126/scitranslmed.aag1711 PMC5550900 27798263

[33] T. Taifour , S. S. Attalla , D. Zuo , Y. Gu , V. Sanguin‐Gendreau , H. Proud , E. Solymoss , T. Bui , H. Kuasne , V. Papavasiliou , C. G. Lee , S. Kamle , P. M. Siegel , J. A. Elias , M. Park , W. J. Muller , Immunity 2023, 56, 2755. 38039967 10.1016/j.immuni.2023.11.002

[34] J. Li , Y. Xia , B. Sun , N. Zheng , Y. Li , X. Pang , F. Yang , X. Zhao , Z. Ji , H. Yu , F. Chen , X. Zhang , B. Zhao , J. Jin , S. Yang , Z. Cheng , Cell Commun. Signaling 2023, 21, 86. 10.1186/s12964-023-01112-5 PMC10152773 37127629

[35] A. Guy , G. Garcia , V. Gourdou‐Latyszenok , L. Wolff‐Trombini , L. Josserand , Q. Kimmerlin , S. Favre , B. Kilani , C. Marty , Y. Boulaftali , S. Labrouche‐Colomer , O. Mansier , C. James , J. Thromb. Haemostasis 2024, 22, 172. 37678548 10.1016/j.jtha.2023.08.028

[36] J. Albrengues , M. A. Shields , D. Ng , C. G. Park , A. Ambrico , M. E. Poindexter , P. Upadhyay , D. L. Uyeminami , A. Pommier , V. Kuttner , E. Bruzas , L. Maiorino , C. Bautista , E. M. Carmona , P. A. Gimotty , D. T. Fearon , K. Chang , S. K. Lyons , K. E. Pinkerton , L. C. Trotman , M. S. Goldberg , J. T. H. Yeh , M. Egeblad , Science 2018, 361, eaao4227. 30262472 10.1126/science.aao4227 PMC6777850

[37] L. Yang , Q. Liu , X. Zhang , X. Liu , B. Zhou , J. Chen , D. Huang , J. Li , H. Li , F. Chen , J. Liu , Y. Xing , X. Chen , S. Su , E. Song , Nature 2020, 583, 133. 32528174 10.1038/s41586-020-2394-6

[38] X. Cheng , H. Zhang , A. Hamad , H. Huang , A. Tsung , Semin. Cancer Biol. 2022, 86, 408. 35066156 10.1016/j.semcancer.2022.01.006 PMC11770836

[39] P. Shen , P. Cheng , Y. Li , G. Zong , R. Deng , C. Qian , Y. Zhao , Z. Wei , Y. Lu , Eur. J. Pharmacol. 2024, 962, 176217. 38036200 10.1016/j.ejphar.2023.176217

[40] Y. Liu , X. Zhang , S. Chen , J. Wang , S. Yu , Y. Li , M. Xu , H. Aboubacar , J. Li , T. Shan , J. Wang , G. Cao , Clin. Mol. Hepatol. 2022, 28, 522. 35508957 10.3350/cmh.2022.0039 PMC9293619

[41] J. Ding , Z. Zhang , W. Huang , G. Bi , Microbiol. Immunol. 2021, 65, 257. 33871094 10.1111/1348-0421.12885

[42] X.‐Y. He , Y. Gao , D. Ng , E. Michalopoulou , S. George , J. M. Adrover , L. Sun , J. Albrengues , J. Daßler‐Plenker , X. Han , L. Wan , X. S. Wu , L. S. Shui , Y.‐H. Huang , B. Liu , C. Su , D. L. Spector , C. R. Vakoc , L. Van Aelst , M. Egeblad , Cancer Cell 2024, 42, 474. 38402610 10.1016/j.ccell.2024.01.013 PMC11300849

[43] M. Yan , Y. Gu , H. Sun , Q. Ge , Front. Immunol. 2023, 14, 1135086. 36993957 10.3389/fimmu.2023.1135086 PMC10040667

[44] C. Kaltenmeier , H. O. Yazdani , K. Morder , D. A. Geller , R. L. Simmons , S. Tohme , Front. Immunol. 2021, 12, 785222. 34899751 10.3389/fimmu.2021.785222 PMC8652262

[45] S. Canè , R. M. Barouni , M. Fabbi , J. Cuozzo , G. Fracasso , A. Adamo , S. Ugel , R. Trovato , F. De Sanctis , M. Giacca , R. Lawlor , A. Scarpa , B. Rusev , G. Lionetto , S. Paiella , R. Salvia , C. Bassi , S. Mandruzzato , S. Ferrini , V. Bronte , Sci. Transl. Med. 2023, 15, eabq6221. 36921034 10.1126/scitranslmed.abq6221

[46] A. Mousset , E. Lecorgne , I. Bourget , P. Lopez , K. Jenovai , J. Cherfils‐Vicini , C. Dominici , G. Rios , C. Girard‐Riboulleau , B. Liu , D. L. Spector , S. Ehmsen , S. Renault , C. Hego , F. Mechta‐Grigoriou , F.‐C. Bidard , M. G. Terp , M. Egeblad , C. Gaggioli , J. Albrengues , Cancer Cell 2023, 41, 757. 37037615 10.1016/j.ccell.2023.03.008 PMC10228050

[47] C. Feng , Y. Li , Y. Tai , W. Zhang , H. Wang , S. Lian , E. E.‐M.‐B.‐K. Jin‐si‐han , Y. Liu , X.LiLi , Q. Chen , M. He , Z. Lu , Sci. Rep. 2023, 13, 19297. 37935721 10.1038/s41598-023-45558-6 PMC10630512

[48] L. Niu , Y. Zhu , M. Wan , C. Wang , X. Hao , J. Song , C. Lei , Z. Qin , F. Tay , L. Niu , Interdiscip. Med. 2024, 2, e20230061.

[49] W. Hu , S. M. L. Lee , A. V. Bazhin , M. Guba , J. Werner , H. Nieß , J. Cancer Res. Clin. Oncol. 2023, 149, 2191. 36050539 10.1007/s00432-022-04310-9 PMC9436160

[50] C. Li , J. Wu , L. Zhang , F. Wang , L. Xu , Y. Zhao , Y. Xiao , F. Zhuang , L. Hou , D. Zhao , Y. She , D. Xie , C. Chen , JTO Clin. Res. Rep. 2023, 4, 100556. 37654895 10.1016/j.jtocrr.2023.100556 PMC10466912

[51] Y. Wan , J. Shen , J. Ouyang , P. Dong , Y. Hong , L. Liang , J. Liu , Front. Immunol. 2022, 13, 1025861. 36341351 10.3389/fimmu.2022.1025861 PMC9634160

[52] a) L. Cristinziano , L. Modestino , A. Antonelli , G. Marone , H.‐U. Simon , G. Varricchi , M. R. Galdiero , Semin. Cancer Biol. 2022, 79, 91; 34280576 10.1016/j.semcancer.2021.07.011

[53] a) M. R. Rios , G. Garoffolo , G. Rinaldi , A. Megia‐Fernandez , S. Ferrari , C. T. Robb , A. G. Rossi , M. Pesce , M. Bradley , Chem. Commun. 2021, 57, 97; 10.1039/d0cc07028a 33332505

[54] Y. Li , R. Yuan , T. Ren , B. Yang , H. Miao , L. Liu , Y. Li , C. Cai , Y. Yang , Y. Hu , C. Jiang , Q. Xu , Y. Zhang , Y. Liu , Cell Death Dis. 2021, 12, 30. 33414368 10.1038/s41419-020-03286-z PMC7791032

[55] W. D. Krautgartner , L. Vitkov , Micron 2008, 39, 367. 17498964 10.1016/j.micron.2007.03.007

[56] R. H. Pires , S. B. Felix , M. Delcea , Nanoscale 2016, 8, 14193. 27387552 10.1039/c6nr03416k

[57] H.‐Y. Kwon , J.‐Y. Kim , X. Liu , J. Y. Lee , J. K. H. Yam , L. Dahl Hultqvist , W. Xu , M. Rybtke , T. Tolker‐Nielsen , W. Heo , J.‐J. Kim , N.‐Y. Kang , T. Joo , L. Yang , S.‐J. Park , M. Givskov , Y.‐T. Chang , Biomater. Sci. 2019, 7, 3594. 31329200 10.1039/c9bm00152b

[58] A. Skallberg , K. Bunnfors , C. Brommesson , K. Uvdal , Anal. Chem. 2019, 91, 13514. 31553180 10.1021/acs.analchem.9b02579

[59] J. S. Holsapple , L. Schnitzler , L. Rusch , T. H. Baldeweg , E. Neubert , S. Kruss , L. Erpenbeck , Biophys. Rep. 2023, 3, 100091. 10.1016/j.bpr.2022.100091 PMC9813678 36619899

[60] C. Petchakup , S. O. Wong , R. Dalan , H. W. Hou , Lab Chip 2023, 23, 3936. 37584074 10.1039/d3lc00398a

[61] A. Muñiz‐Buenrostro , A. Y. Arce‐Mendoza , E. I. Montes‐Zapata , R. C. Calderón‐Meléndez , H. A. Vaquera‐Alfaro , J. A. Huerta‐Polina , M. J. Montelongo‐Rodríguez , Biochem. Biophys. Rep. 2023, 34, 101437. 36817094 10.1016/j.bbrep.2023.101437 PMC9932730

[62] B. Matta , J. Battaglia , B. J. Barnes , Bio‐Protoc. 2023, 13, e4701. 37397793 10.21769/BioProtoc.4701 PMC10308188

[63] F. Liang , J. Zhu , H. Chai , Y. Feng , P. Zhao , S. Liu , Y. Yang , L. Lin , L. Cao , W. Wang , Small Methods 2023, 7, 2201492. 10.1002/smtd.202201492 36950762

[64] Y. Yang , S. Guan , Z. Ou , W. Li , L. Yan , B. Situ , Interdiscip. Med. 2023, 1, e20230013.

[65] M. Jiménez‐Alcázar , C. Rangaswamy , R. Panda , J. Bitterling , Y. J. Simsek , A. T. Long , R. Bilyy , V. Krenn , C. Renné , T. Renné , S. Kluge , U. Panzer , R. Mizuta , H. G. Mannherz , D. Kitamura , M. Herrmann , M. Napirei , T. A. Fuchs , Science 2017, 358, 1202. 29191910 10.1126/science.aam8897

[66] S. Mahri , E. Hardy , T. Wilms , H. De Keersmaecker , K. Braeckmans , S. De Smedt , C. Bosquillon , R. Vanbever , Int. J. Pharm. 2021, 593, 120107. 33259904 10.1016/j.ijpharm.2020.120107

[67] Y. Xia , J. He , H. Zhang , H. Wang , G. Tetz , C. A. Maguire , Y. Wang , A. Onuma , D. Genkin , V. Tetz , A. Stepanov , S. Terekhov , V. Ukrainskaya , H. Huang , A. Tsung , Mol. Oncol. 2020, 14, 2920. 32813937 10.1002/1878-0261.12787 PMC7607180

[68] N. Li , X. Zheng , M. Chen , L. Huang , L. Chen , R. Huo , X. Li , Y. Huang , M. Sun , S. Mai , Z. Wu , H. Zhang , J. Liu , C.‐T. Yang , Clin. Transl. Immunol. 2022, 11, e1386. 10.1002/cti2.1386 PMC9021716 35474906

[69] W. Li , H. Nakano , W. Fan , Y. Li , P. Sil , K. Nakano , F. Zhao , P. W. Karmaus , S. A. Grimm , M. Shi , X. Xu , R. Mizuta , D. Kitamura , Y. Wan , M. B. Fessler , D. N. Cook , I. Shats , X. Li , L. Li , JCI Insight 2023, 8, e168161. 37581941 10.1172/jci.insight.168161 PMC10544201

[70] H. Englert , J. Göbel , D. Khong , M. Omidi , N. Wolska , S. Konrath , M. Frye , R. K. Mailer , M. Beerens , J. C. Gerwers , R. J. S. Preston , J. Odeberg , L. M. Butler , C. Maas , E. X. Stavrou , T. A. Fuchs , T. Renné , Front. Immunol. 2023, 14, 1181761. 37287977 10.3389/fimmu.2023.1181761 PMC10242134

[71] V. Thammavongsa , D. M. Missiakas , O. Schneewind , Science 2013, 342, 863. 24233725 10.1126/science.1242255 PMC4026193

[72] a) K. Nakashima , T. Hagiwara , M. Yamada , J. Biol. Chem. 2002, 277, 49562; 12393868 10.1074/jbc.M208795200

[73] B. Wang , X. Su , B. Zhang , S. Pan , J. Gene Med. 2023, 25, e3530. 37203323 10.1002/jgm.3530

[74] H. Huang , H. Zhang , A. E. Onuma , A. Tsung , Adv. Exp. Med. Biol. 2020, 1263, 13. 32588320 10.1007/978-3-030-44518-8_2 PMC11770835

[75] J. Nishiyama , M. Matsuda , S. Ando , M. Hirasawa , T. Suzuki , H. Makuuchi , Surg. Today 2012, 42, 659. 22200755 10.1007/s00595-011-0105-5

[76] C. Cui , K. Chakraborty , X. A. Tang , G. Zhou , K. Q. Schoenfelt , K. M. Becker , A. Hoffman , Y.‐F. Chang , A. Blank , C. A. Reardon , H. A. Kenny , T. Vaisar , E. Lengyel , G. Greene , L. Becker , Cell 2021, 184, 3163. 33964209 10.1016/j.cell.2021.04.016 PMC10712736

[77] H. Kajioka , S. Kagawa , A. Ito , M. Yoshimoto , S. Sakamoto , S. Kikuchi , S. Kuroda , R. Yoshida , Y. Umeda , K. Noma , H. Tazawa , T. Fujiwara , Cancer Lett. 2021, 497, 1. 33065249 10.1016/j.canlet.2020.10.015

[78] Y. Yin , H. Dai , X. Sun , Z. Xi , J. Zhang , Y. Pan , Y. Huang , X. Ma , Q. Xia , K. He , Clin. Transl. Med. 2023, 13, e1283. 37254661 10.1002/ctm2.1283 PMC10230156

[79] a) K. W. Chen , M. Monteleone , D. Boucher , G. Sollberger , D. Ramnath , N. D. Condon , J. B. von Pein , P. Broz , M. J. Sweet , K. Schroder , Sci. Immunol. 2018, 3, eaar6676; 30143554 10.1126/sciimmunol.aar6676

[80] H. Zhang , X. Xu , R. Xu , T. Ye , Front. Oncol. 2022, 12, 989167. 36132145 10.3389/fonc.2022.989167 PMC9484526

[81] D. Stojkov , M. J. Claus , E. Kozlowski , K. Oberson , O. P. Schären , C. Benarafa , S. Yousefi , H. U. Simon , Sci. Signaling 2023, 16, eabm0517. 10.1126/scisignal.abm0517 36693132

[82] N. Chiang , M. Sakuma , A. R. Rodriguez , B. W. Spur , D. Irimia , C. N. Serhan , Blood 2022, 139, 1222. 34814186 10.1182/blood.2021013422 PMC8612755

[83] a) K. Shirakawa , E. Kobayashi , G. Ichihara , H. Kitakata , Y. Katsumata , K. Sugai , Y. Hakamata , M. Sano , JACC 2022, 7, 146; 35257042 10.1016/j.jacbts.2021.11.005 PMC8897170

[84] H. Yin , H. Lu , Y. Xiong , L. Ye , C. Teng , X. Cao , S. Li , S. Sun , W. Liu , W. Lv , H. Xin , ACS Appl. Mater. Interfaces 2021, 13, 59683. 34902970 10.1021/acsami.1c18660

[85] J. Chen , S. Hou , Q. Liang , W. He , R. Li , H. Wang , Y. Zhu , B. Zhang , L. Chen , X. Dai , T. Zhang , J. Ren , H. Duan , ACS Nano 2022, 16, 2585. 35080858 10.1021/acsnano.1c09318

[86] Y. Hao , X. Li , Y. Liu , D. Liu , X. Zhao , S. Ji , H. Chen , Y. Li , Chem. Eng. J. 2023, 466, 142957.

[87] A. L. C. Penaloza , D. N. Huynh , S. Babity , S. Marleau , D. Brambilla , Mol. Pharmaceutics 2022, 19, 1906. 10.1021/acs.molpharmaceut.2c00086 35543327

[88] L. Zhu , Z. Li , N. Liu , H. Sun , Y. Wang , M. Sun , Adv. Funct. Mater. 2021, 31, 2105089.

[89] D. Zhu , Y. Lu , L. Gui , W. Wang , X. Hu , S. Chen , Y. Wang , Y. Wang , Acta Pharm. Sin. B 2022, 12, 2592. 35646534 10.1016/j.apsb.2021.11.006 PMC9136569

[90] C. E. Galuska , J. A. Dambon , A. Kühnle , K. F. Bornhöfft , G. Prem , K. Zlatina , T. Lütteke , S. P. Galuska , Front. Immunol. 2017, 8, 1229. 29033944 10.3389/fimmu.2017.01229 PMC5626807

[91] H. Liang , Y. Du , C. Zhu , Z. Zhang , G. Liao , L. Liu , Y. Chen , ACS Nano 2023, 17, 2868. 36648411 10.1021/acsnano.2c11280

[92] M. A. Cruz , D. Bohinc , E. A. Andraska , J. Alvikas , S. Raghunathan , N. A. Masters , N. D. van Kleef , K. L. Bane , K. Hart , K. Medrow , M. Sun , H. Liu , S. Haldeman , A. Banerjee , E. M. Lessieur , K. Hageman , A. Gandhi , M. de la Fuente , M. T. Nieman , T. S. Kern , C. Maas , S. de Maat , K. B. Neeves , M. D. Neal , A. Sen Gupta , E. X. Stavrou , Nat. Nanotechnol. 2022, 17, 1004. 35851383 10.1038/s41565-022-01161-w PMC9909445

[93] C. A. Owen , M. A. Campbell , P. L. Sannes , S. S. Boukedes , E. J. Campbell , J. Cell Biol. 1995, 131, 775. 7593196 10.1083/jcb.131.3.775 PMC2120617

[94] N. Filipczak , X. Li , G. R. Saawant , S. S. K. Yalamarty , E. Luther , V. P. Torchilin , J. Controlled Release 2023, 354, 109. 10.1016/j.jconrel.2022.12.062 36596341

[95] Z. Wang , C. Chen , C. Shi , X. Zhao , L. Gao , F. Guo , M. Han , Z. Yang , J. Zhang , C. Tang , C. Zhang , Y. Liu , P. Sun , X. Jiang , J. Controlled Release 2023, 357, 620. 10.1016/j.jconrel.2023.04.013 37061194

[96] L. Han , Y. Zhang , B. Huang , X. Bian , B. Z. Tang , Aggregate 2023, 4, e360.

[97] Y. Li , S. Wu , Y. Zhao , T. Dinh , D. Jiang , J. E. Selfridge , G. Myers , Y. Wang , X. Zhao , S. L. Tomchuck , G. Dubyak , R. T. Lee , B. Estfan , M. Shapiro , S. D. Kamath , A. Mohamed , S. C. C. Huang , A. Y. Huang , R. A. Conlon , S. S. Krishnamurthi , J. R. Eads , J. E. Willis , A. A. Khorana , D. L. Bajor , Z. Wang , J. Clin. Invest. 2024, 134, e175031. 38194275 10.1172/JCI175031 PMC10904055

[98] a) D. M. Gonçalves , S. Chiasson , D. Girard , Toxicol. In Vitro 2010, 24, 1002; 20005940 10.1016/j.tiv.2009.12.007

[99] H. O. Yazdani , C. Kaltenmeier , K. Morder , J. Moon , M. Traczek , P. Loughran , R. Zamora , Y. Vodovotz , F. Li , J. H. C. Wang , D. A. Geller , R. L. Simmons , S. Tohme , Hepatology 2021, 73, 2494. 32924145 10.1002/hep.31552 PMC7956053

[100] L. Tao , M. Xu , X. Dai , T. Ni , D. Li , F. Jin , H. Wang , L. Tao , B. Pan , J. R. Woodgett , Y. Qian , Y. Liu , Oxid. Med. Cell. Longevity 2018, 2018, 4908328. 10.1155/2018/4908328 PMC6120273 30210653

[101] S. Masuda , K. Kato , M. Ishibashi , Y. Nishibata , A. Sugimoto , D. Nakazawa , S. Tanaka , U. Tomaru , I. Tsujino , A. Ishizu , Exp. Mol. Pathol. 2022, 125, 104754. 35259405 10.1016/j.yexmp.2022.104754

[102] Y. Lin , H. Tang , H. Teng , W. Feng , F. Li , S. Liu , Y. Liu , Q. Wei , Int. Immunopharmacol. 2024, 127, 111364. 38101221 10.1016/j.intimp.2023.111364

[103] Y. Cheng , Y. Gong , X. Chen , Q. Zhang , X. Zhang , Y. He , L. Pan , B. Ni , F. Yang , Y. Xu , L. Zhou , Y. Yang , W. Chen , Biomaterials 2022, 284, 121506. 35390709 10.1016/j.biomaterials.2022.121506

[104] H. Zhou , C. Zhu , Q. Zhao , J. Ni , H. Zhang , G. Yang , J. Ge , C. Fang , H. Wei , X. Zhou , K. Zhang , Bioact. Mater. 2024, 39, 14. 38783926 10.1016/j.bioactmat.2024.05.022 PMC11112132

[105] L. Vong , R. Lorentz , A. Assa , M. Glogauer , P. Sherman , J. Immunol. 2014, 192, 1870. 24465012 10.4049/jimmunol.1302286

[106] L. Wei , X. Wang , M. Luo , H. Wang , H. Chen , C. Huang , Hum. Exp. Toxicol. 2021, 40, 1074. 33355008 10.1177/0960327120979028

[107] L. Shi , H. Yao , Z. Liu , M. Xu , A. Tsung , Y. Wang , Mol. Cancer Res. 2020, 18, 735. 32193354 10.1158/1541-7786.MCR-19-0018 PMC7668292

[108] H. Deng , C. Y. Lin , L. Garcia‐Gerique , S. Y. Fu , Z. Cruz , E. E. Bonner , M. Rosenwasser , S. Rajagopal , M. N. Sadhu , C. Gajendran , M. Zainuddin , R. Gosu , D. Sivanandhan , M. A. Shelef , B. Nam , D. T. Vogl , D. I. Gabrilovich , Y. Nefedova , Cancer Res. 2022, 82, 3561. 36069973 10.1158/0008-5472.CAN-21-4045 PMC9532374

[109] M. Li , C. Lin , H. Deng , J. Strnad , L. Bernabei , D. T. Vogl , J. J. Burke , Y. Nefedova , Mol. Cancer Ther. 2020, 19, 1530. 32371579 10.1158/1535-7163.MCT-19-1020 PMC7335350

[110] Z. Wu , G. Lu , L. Zhang , L. Ke , C. Yuan , N. Ma , X. Yu , X. Guo , W. Zhao , Y. Wang , S. Hu , D. Wu , W. Li , Int. Immunopharmacol. 2021, 94, 107486. 33639566 10.1016/j.intimp.2021.107486

[111] a) Y. Wada , K. Yoshida , J. Hihara , K. Konishi , K. Tanabe , K. Ukon , J. Taomoto , T. Suzuki , H. Mizuiri , Cancer Sci. 2006, 97, 1037; 16918998 10.1111/j.1349-7006.2006.00278.x PMC11158772

[112] H. Zhao , Y. Liang , C. Sun , Y. Zhai , X. Li , M. Jiang , R. Yang , X. Li , Q. Shu , G. Kai , B. Han , Int. J. Mol. Sci. 2022, 23, 15180. 36499502 10.3390/ijms232315180 PMC9736467

[113] J. Zeng , H. Xu , P. Z. Fan , J. Xie , J. He , J. Yu , X. Gu , C. J. Zhang , J. Cell. Mol. Med. 2020, 24, 7590. 32427405 10.1111/jcmm.15394 PMC7339206

